# Molecular Characterization of Multidrug-Resistant and Extended-Spectrum β-Lactamases-Producing *Salmonella enterica* Serovars Enteritidis and Typhimurium Isolated from Raw Meat in Retail Markets

**DOI:** 10.3390/antibiotics13070586

**Published:** 2024-06-24

**Authors:** Md. Mahfujur Rahman, Hemayet Hossain, Md. Shahidur Rahman Chowdhury, Md. Mukter Hossain, Asmaa Saleh, Reem Binsuwaidan, Ayman Noreddin, Yosra A. Helmy, Mohamed E. El Zowalaty

**Affiliations:** 1 Department of Medicine, Faculty of Veterinary, Animal and Biomedical Sciences, Sylhet Agricultural University, Sylhet 3100, Bangladesh; shahidur.vetmed@sau.ac.bd (M.S.R.C.); mukter.vetmed@sau.ac.bd (M.M.H.); 2 Department of Anatomy and Histology, Faculty of Veterinary, Animal and Biomedical Sciences, Sylhet Agricultural University, Sylhet 3100, Bangladesh; hemayet.vabs@student.sau.ac.bd; 3 Department of Pharmaceutical Sciences, College of Pharmacy, Princess Nourah Bint Abdulrahman University, P.O. Box 84428, Riyadh 11671, Saudi Arabia; asali@pnu.edu.sa (A.S.);; 4 Department of Clinical Pharmacy, Faculty of Pharmacy, Ahram Canadian University, 6th of October City 3221405, Egypt; 5 Department of Veterinary Science, Martin-Gatton College of Agriculture, Food, and Environment, University of Kentucky, Lexington, KY 40546, USA; 6 Department of Zoonoses, Faculty of Veterinary Medicine, Suez Canal University, Ismailia 41522, Egypt; 7 Department of Microbiology and Immunology, Faculty of Pharmacy, Ahram Canadian University, 6th of October City 3221405, Egypt

**Keywords:** *S.* Typhimurium, *S.* Enteritidis, multidrug resistance, extended-spectrum β-lactamases, Bangladesh, chicken, livestock, cattle, meat

## Abstract

In the present study, a total of 720 samples were collected from retail raw meat from 13 upazilas in Sylhet District, Bangladesh, of which 225 samples were from cattle meat, 210 samples were from goat meat, and 285 samples were from chicken meat. *Salmonella enterica* serovars Typhimurium and Enteritidis were screened for extended-spectrum β-lactamase (ESBL) genes using multiplex PCR. Among the 720 samples, *Salmonella* spp. was detected in 28.06% (202 out of 720) of the samples, with *S.* Enteritidis and *S.* Typhimurium were identified in 11.53% (83 out of 720) and 12.22% (88 out of 720) of the samples, respectively. It was found that all *Salmonella enterica* serovars isolated from cattle meat displayed multidrug resistance (MDR) based on antimicrobial susceptibility testing. Notably, a significant proportion of *S.* Enteritidis isolates and all *S.* Typhimurium isolates from goat meat demonstrated complete resistance to multiple drugs (ampicillin, cefuroxime, and ceftazidime). Regarding chicken meat, out of 89 isolates encompassing both *S.* Typhimurium and *S.* Enteritidis, 57 isolates (64.04%) exhibited MDR. Additionally, *bla*_CTX-M-1_ exhibited the highest occurrence at 15.69% for *S.* Typhimurium and 7.89% for *S.* Enteritidis in chicken meat. Moreover, *bla*_CTX-M-9_ was only detected at 3.92% for *S.* Enteritidis in chicken meat. Furthermore, *bla*_OXA_ had the highest prevalence rate of 19.04% for *S.* Enteritidis and 25.80% for *S.* Typhimurium in cattle meat, followed by chicken meat. These findings highlight the urgency for monitoring ESBL-producing *Salmonella* in retail raw meat and the need for strict measure to manage antibiotic use to prevent the spread of multidrug-resistant and ESBL-producing *Salmonella* strains, thereby protecting humans and reducing public health risks.

## 1. Introduction

*Salmonella* remains a leading cause of bacterial foodborne illnesses worldwide [1,2] contributing to approximately 10% of global mortality, predominantly originating from animal sources, resulting in an estimated 33 million deaths annually [3]. Salmonellosis is one of the most common zoonotic diseases, and *Salmonella* is a common cause of foodborne disease outbreaks. The prevalence of *Salmonella* spp. infection is a significant public health issue and a continuous threat worldwide [4]. The incidence of *Salmonella* infections significantly increases the global burden of gastroenteritis, with an estimated 93.8 million cases reported annually, leading to 155,000 deaths [5]. Gram-negative bacteria such as *Salmonella* employ β-lactamases as a primary defense mechanism against β-lactam antibiotics. The emergence of extended-spectrum β-lactamases (ESBLs) poses a significant global threat, with over 300 distinct types identified [6].

Antimicrobial resistance is a critical global health and development threat, largely driven by the indiscriminate use and overuse of antimicrobial agents in both food animals and humans [7,8]. Countries such as Bangladesh face increased risks of antimicrobial resistance due to significant problems hindering the implementation of antibiotic stewardship, limited regulatory programmed surveillance, and limited monitoring systems regarding antimicrobial use and resistance prevention [9]. Antimicrobial agents are commonly used in cattle, goats, and chickens for disease prevention and treatment, with many of these drugs also being frequently used in human medicine, contributing to potential antimicrobial resistance in both animals and humans [10]. A major risk of zoonotic infections is the spillover of multidrug-resistant *Salmonella* strains from livestock to humans via food chain including contaminated food, water, direct contact, or the ingestion of contaminated materials derived from infected livestock. Given these concerns, it is imperative to detect *Salmonella* enterica in livestock meat especially cattle, goat, and chicken and investigate their multidrug resistance (MDR) profiles.

Over the past few decades, *S.* Typhimurium and *S.* Enteritidis have become major causes of global salmonellosis outbreaks [11]. The economic impact of *Salmonella* infections has gained increased attention in developed countries. Animals are exposed to *Salmonella* through various means, including water, feed, feces, soil, and insects, becoming infected or serving as asymptomatic carriers. Cattle, goats, and chicken meat are significant animal-origin protein sources which are widely consumed in Bangladesh and globally [12,13,14]. However, in many resource-limited regions, these animals are slaughtered in small, unhygienic abattoirs, facilitating microbial contamination, survival, and spread to the surrounding environment and food handlers [15,16,17,18,19]. Several studies have reported the prevalence of *Salmonella* in meat, with detection rates ranging from 6.79% to 97.6% in chicken meat in India [20] and 21.1% in Bangladesh [21]. Moreover, the prevalence of *Salmonella enterica* serovar in cattle meat was reported to be 23.3% in Egypt [22], 64.28% in Pakistan [23], 23% in Nigeria [24], 30.55% in Morocco [25], and 29.8% in Tunisia [26]. The reported prevalence rates of *Salmonella* in goat meat varies globally, with rates as high as 60% reported in Pakistan [23]. The detection of *Salmonella* was reported from goat meat swabs in different regions of different countries such as a rate of 8.3% in Modjo (Ethiopia), 7.5% in Bishoftu (Ethiopia), 4% in Arusha (Tanzania), and 3.5% in Gujrat (India) [27,28,29].

Antimicrobial resistance in livestock production systems including common meat-producing animals such as cattle, goat, and chicken, particularly multidrug-resistant *Salmonella enterica* serovars Enteritidis and Typhimurium, remains largely unexplored in Bangladesh. Recent studies have highlighted the significant detection of MDR *E. coli* in commercial cattle, goats, and poultry [30,31,32]. Thus, the current study aimed to determine and compare the prevalence and antimicrobial resistance of ESBL-producing and MDR *Salmonella enterica* serovars Enteritidis and Typhimurium in retail meat samples in Bangladesh.

## 2. Results

### 2.1. Molecular Detection of Salmonella enterica Serovars

**Figure 1:**
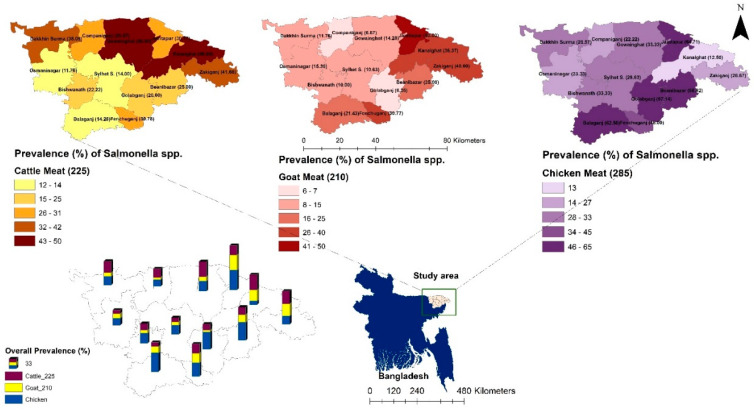
The location (sub-district) and the prevalence of *Salmonella* spp. isolated from samples of retail cattle, goat, and chicken meat in Sylhet District, Bangladesh. Bangladesh country map is shown in blue and the box indicates the selected study area in the present study.

**Figure 2:**
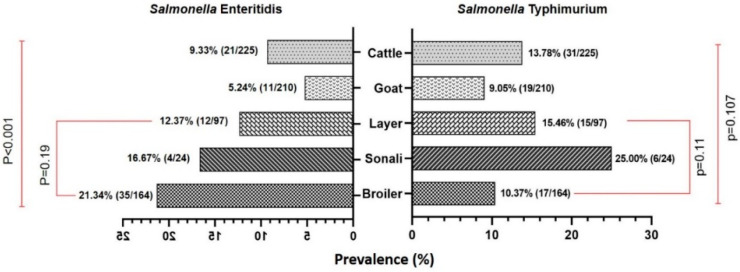
A bi-directional bar (Mirror bar) diagram showing the prevalence of *Salmonella enterica* serovar Enteritidis and Typhimurium in cattle and poultry retail meat samples compared to goat meat samples. (χ^2^-test, Level of Significance *p* < 0.05).

Out of 720 samples, the prevalence of *Salmonella* spp. was found to be 28.06% (202 out of 720; 95% CI: 24.80–31.49). Specifically, the prevalence rate of *S.* Enteritidis was 11.53% (83 out of 720; 95% CI: 9.29–14.09), while the prevalence rate of *S.* Typhimurium was 12.22% (88 out of 720; 95% CI: 9.92–14.84). Out of 225 retail cattle meat samples, the prevalence rates of *Salmonella* spp., *S.* Typhimurium, and *S.* Enteritidis were 26.22%, 13.78%, and 9.33%, respectively (as illustrated in Figure 1 and Figure 2).

Analysis of the prevalence of *Salmonella* spp. across different upazilas within the district revealed that both the Kanaighat and Gowainghat Upazilas had the highest prevalence rate (50.00%) of *Salmonella* spp., while Osmaninagar displayed the lowest rate of 11.76% (Figure 1). Moreover, Gowainghat Upazila exhibited the highest prevalence of *S.* Typhimurium in retail cattle meat samples, accounting for 35.71%, whereas Osmaninagar Upazila showed the lowest prevalence rate of 5.88% (Table 1).

Similarly, Dakkhin Surma Upazila demonstrated the highest prevalence of *S.* Enteritidis in retail cattle meat samples, accounting for 23.81%, whereas only 2.00% of the samples collected from Sylhet Sadar Upazila were *S.* Enteritidis positive (Table 2).

In the present study, the prevalence of *Salmonella* spp. in chicken meat samples was 36.84% (105/285) which was found to be higher than that of the prevalence rates in cattle and goat meat samples. Of these isolates, 51 (17.89%) were found to be *S.* Enteritidis, and 38 (13.33%) were found to be *S.* Typhimurium. Jaintapur Upazila had the highest prevalence (64.71%) of *Salmonella* spp., while Kanaighat displayed the lowest (12.50%) (Figure 1). Balaganj Upazila exhibited the highest prevalence of *Salmonella* Typhimurium, accounting for 25.00% (9.77–46.71%), while Companiganj Upazila showed the lowest prevalence at 5.56% (0.14–27.29%) (Table 1). Similarly, Golapganj Upazila demonstrated the highest (35.71%) prevalence of *S.* Enteritidis in chicken meat samples, whereas no positive samples were found in Kanaighat Upazila (Table 2). Moreover, the specific prevalence rates of *S.* Typhimurium in various types of retail chicken meat samples, with broiler, sonali, and layer meats, exhibited prevalence rates of 10.37% (17/164), 25.00% (6/24), and 15.46% (15/97), respectively (Figure 2). Similarly, the prevalence rates for *S.* Enteritidis in different types of retail chicken meats, with broiler, sonali, and layer meats, demonstrated prevalence rates of 21.34% (35/164), 16.67% (4/24), and 12.37% (12/97), respectively (Figure 2).

The prevalence rate of *Salmonella* spp. isolated from samples obtained from retail goat meat (Figure 1) was recently reported [33], and it was found to be 18.10% (38/210; 95% CI: 13.13–23.98), which was lower than the prevalence rates of *Salmonella* spp. in both cattle and meat samples reported in the present study. The highest prevalence was observed in Jaintapur at 50% (6 out of 12), followed by Zakiganj at 40% (4 out of 10). The lowest prevalence was reported in Golapganj at 6.25% (1 out of 16) as previously reported [33]. The distribution of the prevalence of *Salmonella enterica* serovars in retail goat meat samples across different upazilas of Sylhet District compared to cattle and chicken samples is shown in Table 1 and Table 2. The investigation also revealed that the prevalence rates of *S.* Typhimurium and *S.* Enteritidis were 9.05% (95% CI: 5.54–13.77) and 5.24% (95% CI: 2.64–9.18), respectively (Figure 2 and Table 1 and Table 2). The detailed geographical distribution of these prevalence rates is provided in Table 1 and Table 2.

### 2.2. Antimicrobial Resistance and Phenotypic Correlations

**Figure 3:**
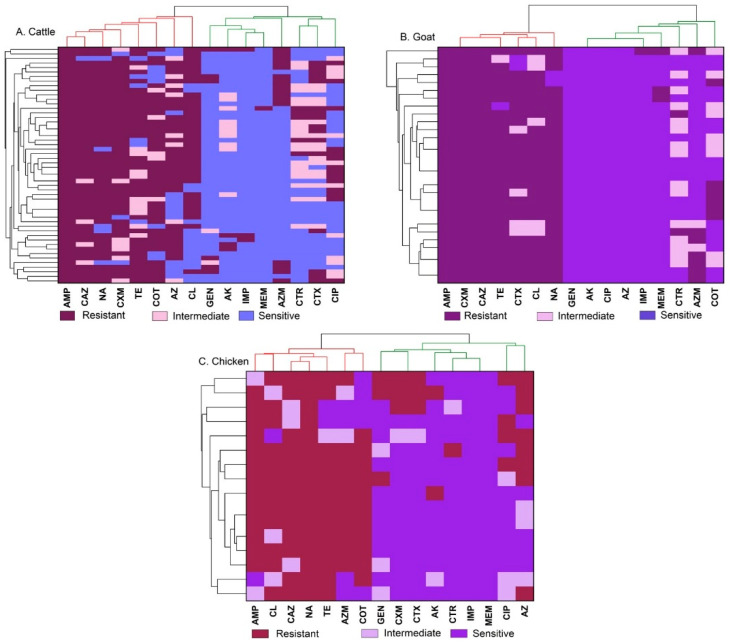
A heatmap showing the sensitivity pattern of antibiotics with clustering as a dendrogram. The clustering of antibiotics was represented by a dendrogram for cattle (**A**), goat (**B**), and chicken (**C**) meat samples. Ampicillin (AMP), gentamicin (GEN), amikacin (AK), cefuroxime (CXM), ceftriaxone (CTR), cefotaxime (CTX), ceftazidime (CAZ), meropenem (MEM), imipenem (IMP), tetracycline (TE), ciprofloxacin (CIP), azithromycin (AZ), aztreonam (AZM), chloramphenicol (CL), sulfamethoxazole-trimethoprim (COT), and nalidixic acid (NA).

A heatmap was generated using hierarchical cluster analysis (HCA) and illustrated the antibiotic susceptibility pattern. The clustering of antibiotics was represented by a dendrogram (Figure 3A–C).

The majority of isolates from cattle meat samples exhibited resistance to ampicillin (100%), ceftazidime, nalidixic acid, among others, while demonstrating high sensitivity to meropenem and imipenem (Figure 3A). Additionally, *Salmonella* Typhimurium and *Salmonella* Enteritidis obtained from goat meat samples (Figure 3B) displayed complete resistance to ampicillin, ceftazidime and cefuroxime while 100% sensitive to amikacin, ciprofloxacin and gentamicin. Among *Salmonella* Enteritidis isolates from chicken meat (Figure 3C), 100% showed resistance to nalidixic acid while demonstrating the highest sensitivity to meropenem, imipenem, and ceftriaxone. Similarly, all *Salmonella* Typhimurium isolates from chicken meat samples displayed resistance to ampicillin with the isolates demonstrated the highest sensitivity to meropenem, imipenem, and amikacin.

**Figure 4:**
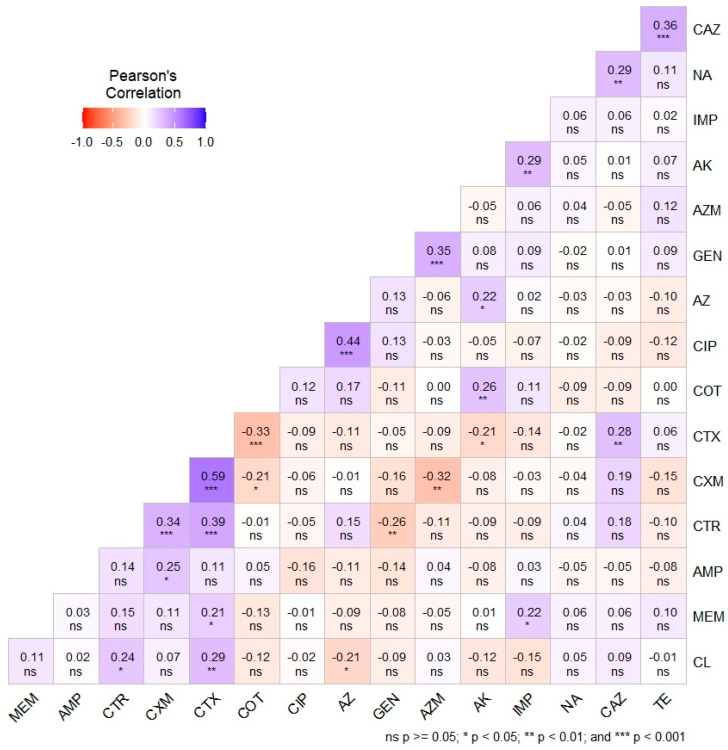
Pearson’s correlation coefficient showing the phenotypic correlation among the antimicrobial agents with levels of significance. Ampicillin (AMP), gentamicin (GEN), amikacin (AK), cefuroxime (CXM), ceftriaxone (CTR), cefotaxime (CTX), ceftazidime (CAZ), meropenem (MEM), imipenem (IMP), tetracycline (TE), ciprofloxacin (CIP), azithromycin (AZ), aztreonam (AZM), chloramphenicol (CL), sulfamethoxazole-trimethoprim (COT), and nalidixic acid (NA).

Pearson’s correlation coefficient analysis (Figure 4) depicts the phenotypic correlation between antimicrobial agents, along with the level of significance. In addition, Figure 4 illustrates the phenotypic correlation coefficients (r) among the selected antibiotics, represented by a spectrum ranging from light to deep violet for positive correlations (0 to +1) and light to deep red for negative correlations (0 to −1). A moderately strong positive correlation was observed between CXM and CTX (r = 0.59, *p* < 0.001). Additionally, moderate positive correlations were noted between CIP and AZ (r = 0.44, *p* < 0.001), as well as between TE and CAZ (r = 0.36, *p* < 0.001). Conversely, significantly weak positive correlations were found between CXM and CTR, AZM and GEN, and CTX and CTR, among others. Weak negative correlations were observed between CTX and COT (r = −0.33, *p* < 0.001), AZM and CXM (r = −0.32, *p* < 0.01), and CTR and GEN (r = −0.26, *p* < 0.01), among others. The majority of the relationships were characterized by very weak positive and negative correlations, with further details depicted in Figure 4.

### 2.3. Antibiogram Profile of S. enterica Serovar Typhimurium and Enteritidis

**Figure 5:**
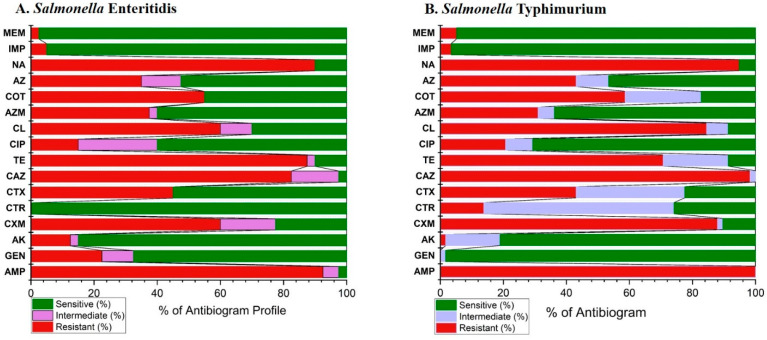
The overall antibiogram profiles of *S. enterica* Serovar Typhimurium and Enteritidis isolates in the present study. Ampicillin (AMP), gentamicin (GEN), amikacin (AK), cefuroxime (CXM), ceftriaxone (CTR), cefotaxime (CTX), ceftazidime (CAZ), meropenem (MEM), imipenem (IMP), tetracycline (TE), ciprofloxacin (CIP), azithromycin (AZ), aztreonam (AZM), chloramphenicol (CL), sulfamethoxazole-trimethoprim (COT), and nalidixic acid (NA).

The comprehensive antibiogram profiles of *S. enterica* Serovar Typhimurium and Enteritidis isolates are shown in Figure 5 and detailed in Supplementary Tables S1 and S2. *S.* Enteritidis exhibited the highest resistance to ampicillin (92.5%), followed by nalidixic acid (90%), and tetracycline (87.5%). Conversely, all isolates were sensitive to ceftriaxone (100%), followed by meropenem (97.5%), and imipenem (95%). *S.* Typhimurium showed complete resistance to ampicillin and a high resistance to ceftazidime (98.28%). In contrast, isolates were generally sensitive to gentamicin (98.28%), imipenem (96.55%) and meropenem (94.83%) among the 16 tested antimicrobial agents.

### 2.4. MAR Index and MDR Profile

**Figure 6:**
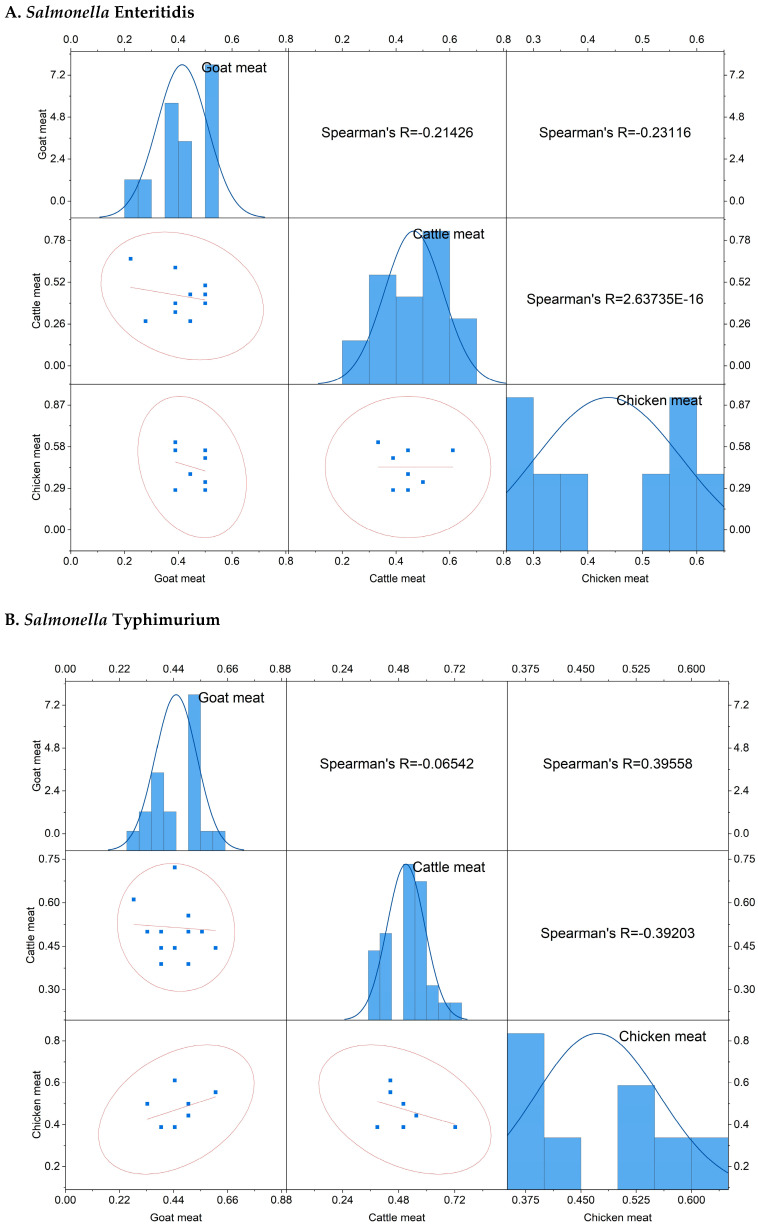

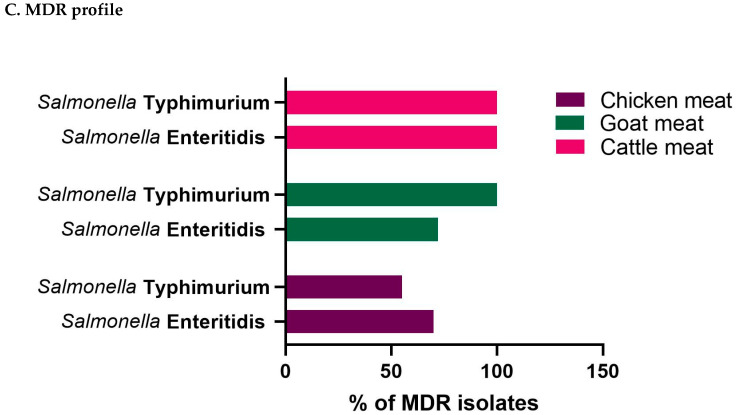
The scatter plot correlation matrix with a histogram showing the multiple antibiotic resistance index of *Salmonella enterica* serovars (**A**) *S.* Enteritidis, (**B**) *S.* Typhimurium, and (**C**) the multidrug resistance (MDR) profiles of the isolates in different livestock (cattle, goat, and chicken) meat samples investigated in the present study.

The scatter plot correlation matrix, along with histograms, illustrates the status and correlation of the MARI of *Salmonella enterica* serovars Enteritidis and Typhimurium among cattle, goat, and chicken meat samples (Figure 6A,B).

The MARI of *S.* Enteritidis isolated from goat meat samples ranged from 0.22 to 0.50 (mean: 0.41), however MARI ranged from 0.28–0.67 (mean: 0.47) in cattle meat samples, and 0.28–0.61 (mean: 0.44) in chicken meat samples. Regarding *S.* Typhimurium, the MARI ranged from 0.28 to 0.61 (mean: 0.45) in goat meat samples, 0.39–0.72 (mean: 0.51) in cattle meat samples, and 0.39–0.61 (mean: 0.47) in chicken meat samples. The MARI value of *S.* Enteritidis isolated from goat meat samples exhibited a weak negative correlation with both cattle and chicken meat samples. Conversely, the MARI value of *S.* Typhimurium in goat meat showed a negligibly negative correlation with cattle meat but a moderately positive correlation (r = 0.396) with chicken meat samples. Additionally, a moderately negative correlation (r = −0.39) was observed between cattle and chicken meat samples.

The MDR profiles of *S.* Typhimurium and *S.* Enteritidis originating from cattle, goat, and chicken meat samples are illustrated in Figure 6C. It was observed that all *Salmonella enterica* serovars isolated from cattle meat samples displayed MDR. Notably, a significant proportion of *S.* Enteritidis isolates (72.73%; 8/11; 95% CI: 39.03–93.98) and all *S.* Typhimurium isolates (100%; 95% CI: 82.35–100.00) from goat meat demonstrated complete resistance to multiple drugs as was previously reported (34). Regarding chicken meat samples, out of 89 isolates including both *S.* Typhimurium (55.26%; 39.45–71.07) and *S.* Enteritidis (70.59%; 58.08–83.09), 57 isolates (64.04%) exhibited MDR.

### 2.5. Molecular Detection of ESBL Genes

In the current study, we screened the presence of ESBL genes in *Salmonella enterica* serovars isolated from cattle, goat, and chicken meat samples obtained from retail meat (Table 3).

In the present study, significantly elevated frequencies of ESBLs, particularly associated with the *bla*_TEM_ genes, were detected using multiplex PCR in both *S.* Typhimurium (70.96%) and *S.* Enteritidis (76.19%) isolates (*p* < 0.001) obtained from cattle meat samples, followed by goat meat and chicken meat samples. The PCR results showed that *bla*_SHV-1_ was the predominant ESBL gene identified among *Salmonella* isolates with 14.28% of *S.* Enteritidis isolates from cattle meat samples and 21.05% of *S.* Typhimurium isolates from goat meat samples. Additionally, *bla*_CTX-M-1_ exhibited the highest occurrence at 15.69% for *S.* Enteritidis and 7.89% for *S.* Typhimurium in chicken meat samples, while not detected in both cattle and goat meat samples in the present study. Moreover, *bla*_CTX-M-9_ was only detected at 3.92% for *S.* Enteritidis in chicken meat samples, with no detection in *S.* Typhimurium and it was not detected in cattle and goat meat samples. Furthermore, *bla*_OXA_ had the highest prevalence at 19.04% for *S.* Enteritidis and 25.80% for *S.* Typhimurium in cattle meat samples, followed by chicken meat samples, while not detected in goat meat samples.

## 3. Discussion

The findings presented in the current study shed light on several important aspects of *Salmonella* contamination, antimicrobial resistance, MDR, and the presence of ESBL genes in the retail meat obtained from livestock in Sylhet District, Bangladesh. The findings have significant implications for public health, food safety, and antimicrobial stewardship efforts.

The prevalence rates of *Salmonella* spp., particularly *S.* Enteritidis and *S.* Typhimurium, in retail meat samples from cattle, goats, and chickens are increasingly alarming. The high prevalence rates indicate a widespread contamination of meat products, posing a significant risk to consumers. These findings highlight the urgent need for improved hygienic practices during meat production, processing, and handling to mitigate the risk of *Salmonella* contamination.

In the current study, the prevalence of *Salmonella* spp. in retail cattle meat samples in Sylhet District, Bangladesh was 26.22%, which was closely similar to findings from studies in Egypt (23.3%), Pakistan (21.8%), and Nigeria (23%) [22,23,24]. Notably, the prevalence of *Salmonella enterica* in the current study was lower than that reported in previous studies in Morocco (30.55%), Tunisia (29.8%), and Vietnam (64.1%) [25,26,34], while being higher than those documented in Turkey (20%), Egypt (8.8%), Iran (4.35%), and Ecuador (11.47%) [35,36,37,38]. These variations in prevalence can be attributed to differences in sampling size, identification methods, and the selection of target genes.

The findings of the present study demonstrated that *S.* Typhimurium is the dominant serovar among other *Salmonella* spp. identified in retail cattle meat samples, consistent with findings from previous studies [22,34,36,37]. The prevalence also varied among different upazilas, with the highest prevalence recorded at 50% in Kanaighat and Gowainghat, and the lowest prevalence at 11.76% in Osmaninagar. The prevalence of *Salmonella* in goat meat was determined to be 18.10%, which was lower than the findings reported in a study from Pakistan [23] and higher than those from studies conducted in India, Modjo, Bishoftu, Arusha (Tanzania), and Gujarat (India) based on goat carcass swabs [27,28,29]. In the present study, *S.* Typhimurium was identified in cattle meat as the dominant serovar among other *Salmonella* spp. found in retail goat meat, consistent with findings from previous study [30].

The overall prevalence of *Salmonella* spp. was found to be 36.84%, a rate comparable to findings from studies conducted in Spain (35.83%) and Russia (38.5%) in chicken meat [39,40]. However, this prevalence rate was higher than the rates reported from Bangladesh, India, Iran, Vietnam, Trinidad, and Malaysia [41,42,43,44,45,46]. *Salmonella* spp. was detected in various types of retail chicken meat samples, including broiler, spent hen, and sonali, with both *S.* Typhimurium and *S.* Enteritidis being identified, consistent with global research findings. Studies conducted in different regions of Bangladesh have also shown the presence of *S.* Typhimurium and *S.* Enteritidis in commercial broiler, layer, and breeder farms [41,47]. Similar serotypes of *S.* Typhimurium and *S.* Enteritidis have been reported in raw chicken meat sold in Iranian supermarkets [4]. In Turkey, *S.* Enteritidis (21.9%) and *S.* Typhimurium (9.4%) were found to be the most prevalent serotypes in poultry [48]. *Salmonella enterica* serovars have also been identified in backyard chicken flocks in India [49]. Moreover, in Egypt, both *S.* Enteritidis and *S.* Typhimurium were detected in chicken meat [50]. In the present study, it was also found that the prevalence rate of *S.* Typhimurium in sonali chicken meat was notably higher, at 25%, while *S.* Enteritidis was higher in broiler meat, at 21.34%. These findings differ somewhat from other studies conducted in Bangladesh, where the highest prevalence rates were observed in broiler and layer chickens [51,52]. These variations in the prevalence rates could be attributed to factors such as seasonal changes, environmental conditions, or the lack of adequate farm biosecurity measures. Furthermore, *S.* Enteritidis emerged as the dominant serovar in retail chicken meat samples, a trend consistent with research findings from Iran, where *S.* Enteritidis was prevalent in both retail chicken carcasses and meat in Turkey [48,53]. Analysis of the prevalence of salmonellosis in chicken meat across different sampling sites revealed that Jaintapur Upazila had the highest prevalence of 64.71% of *Salmonella* spp., while Kanaighat displayed the lowest prevalence of 12.50%. These discrepancies regarding salmonellosis in retail cattle, goat, and chicken meat samples in the present study may be explained due to various factors including the method of sample collection, the cleanliness of the farms, or the personal hygienic practices during meat processing and food handlers [36,54]. The varying prevalence rates could also be influenced by the sampling of meat from wet markets, where cattle, goats, and chickens may be eviscerated in contaminated areas with intestinal contents and slaughtered under unhygienic conditions [55]. Additionally, discrepancies may arise from differences in the hygiene practices in the retail shops, the awarenesses of food handlers and workers about contamination risks, the infrastructure and diameters of the shops, and urban versus rural location of the markets [56]. It is important to note that in numerous instances, at the time of the current study, no designated slaughterhouse was pinpointed within the retail meat market. Instead, meat vendors frequently slaughter cattle, goats, and poultry near their shops, increasing the likelihood of meat contamination with environmental pathogenic microorganisms. In Sylhet District, Bangladesh, a notable practice involves selling the guts and intestines of slaughtered animals alongside the meat, posing an additional considerable risk of contamination by enteric microorganisms such as *Salmonella*.

The antibiotic resistance patterns of *Salmonella enterica* in the present study are closely aligned with those of previous studies [36,46,57,58]. In contrast to previous findings [59], the findings of the current study showed high sensitivity of *Salmonella* Typhimurium and *Salmonella* Enteritidis to gentamicin, ceftriaxone, meropenem, and imipenem, consistent with other reports [46,57,60,61,62].

It was noted that every *Salmonella enterica* serovar isolated from cattle meat exhibited multidrug resistance (MDR), while a significant majority of *S.* Enteritidis isolates (72.73%; 8/11) and all *S.* Typhimurium isolates (100%) from goat meat demonstrated complete resistance to multiple drugs. The lower rates of MDR phenotype between *S.* Typhimurium (55.26%) and *S.* Enteritidis (70.59%) isolated from chicken meat, compared to those from cattle and goat meat, could be attributed to several factors. One plausible explanation is the shorter rearing time of chickens, typically five to seven weeks, compared to cattle and goats. This shorter period may result in less exposure to antimicrobial agents, leading to a lower MDR phenotype in chicken-origin isolates. Additionally, differences in the management practices, such as the use of antimicrobial agents in feed or water, may vary between poultry and livestock production systems, contributing to differences in MDR prevalence among bacterial isolates. Moreover, genetic factors within bacterial populations and selective pressure exerted by antimicrobial usage patterns could also influence an MDR status. These findings are closely aligned with those of a study conducted on Egyptian buffalo meat, which reported MDR rate of 79.2% in *Salmonella* isolates [63]. However, findings of the present study are different from those of another study in Pakistan, where only 11 (10.0%) isolates were identified as MDR [23]. Similarly, a study in South Africa demonstrated that 43% of isolates from livestock and poultry were resistant to multiple drugs [64]. The findings regarding cattle meat isolates are consistent with those of another study where all isolates were MDR [65].

The findings of the present study revealed notably increased frequencies of ESBL genes, particularly associated with the *bla*_TEM_ genes, which aligns with previous studies where *bla*_TEM_ were predominantly detected in *Salmonella* and *Escherichia coli* isolated from poultry and food products of animal origin [36,66,67]. The current findings revealed a lower prevalence of *bla*_SHV_ compared to a previous study which reported a rate of 64% *bla*_SHV_ in *Salmonella* serovars in poultry meat products [68].

Additionally, *bla*_CTX-M-1_ exhibited occurrences, with 15.69% for *S.* Enteritidis and 7.89% for *S.* Typhimurium in chicken meat, which are closely similar to those of previous reports [69,70]. Recent studies reported that 2.7% of *S. enterica* isolates in chicken meat in Bangladesh were positive for *bla*_CTX-M-1_ [65], and *bla*_CTX-M-1_ detection rate of 3.2% was reported for *S.* Heidelberg isolated from poultry production chain in Brazil [71], which partially supports the findings in the current study. Moreover, *bla*_CTX-M-9_ was detected at 3.92% for *S.* Enteritidis in chicken meat samples in the present study. The *bla*_OXA_ exhibited the highest prevalence, at 19.04% for *S.* Enteritidis and 25.80% for *S.* Typhimurium in cattle meat samples, followed by chicken meat samples, which align with a previous study [72]. The variation in the prevalence of ESBL genes, such as *bla*_TEM_, *bla*_SHV_*,* and *bla*_CTX-M-1_, require further investigations in the context of horizontal gene transfer mechanisms, including integrons and gene cassettes which are among the limitations of the present study. However, integrating this aspect into the study findings and future investigations using whole genome sequencing analyses will help provide significant in-depth genomic insights into the potential mechanisms driving the dissemination of antimicrobial resistance determinants among ESBL-producing *Salmonella* strains isolated from non-human livestock origin meat products in retail meat sources in Bangladesh.

## 4. Materials and Methods

### 4.1. Study Design, Site, and Sampling Method

**Figure 7:**
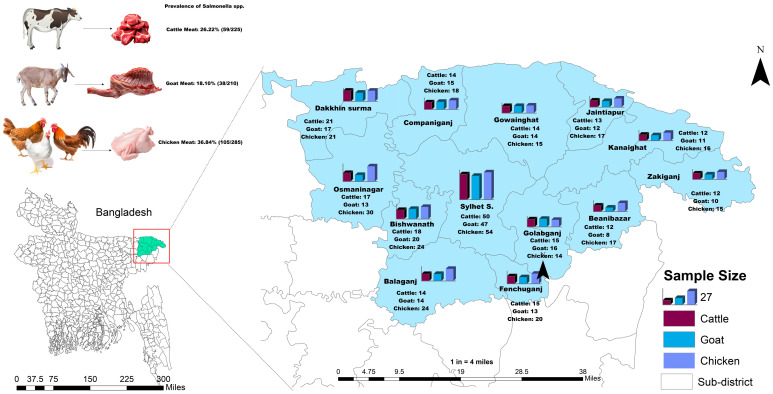
A geographic map of the study area showing the location of the selected study sites and the sample size of retail cattle, goat, and chicken meat samples in Sylhet District, Bangladesh. The map was created using ArcMap 10.7, ESRI, CA, USA.

A cross-sectional investigation was undertaken across 13 sub-districts located in the Sylhet (3452 km^2^), Bangladesh. The selected sub-districts were visualized in Figure 7. Geographically, these upazilas spanned from 24°36′ to 25°11′ North latitude and 91°38′ to 92°30′ East longitude, as shown in Figure 7. Sample procurement followed a convenient sampling approach, based on the accessibility of retail outlets vending cattle, goat, and chicken meat.

### 4.2. Determination of Sample Size

An appropriate number of samples for prevalence calculation was determined using a mathematical formula as previously reported [73]

$$n=\frac{Z^{2}Pexp\left(1-Pexp\right)}{d^{2}}$$

where,

P_exp_ =Expected proportion in population

n=Number of samples

d=Margin of error (0.05)

$Z=z-score\left(1.96\right)$

According to previously published data, the prevalence of *Salmonella* spp. in meat sold at retail outlet was 51.35% [23]. According to the prior study, Pexp = 0.5135 was utilized to optimize the number of samples. Performing the formula, the minimum number of samples was calculated to be 383.88~384. The current investigation included 720 swabs obtained from retail meat samples from 13 upazilas in Sylhet District.

### 4.3. Sample Collection and Bacterial Isolation

Seven hundred and twenty samples obtained from meat swabs were collected, comprising 225 samples from cattle meat, 210 samples from goat meat, and 285 samples from chicken meat, respectively. The livestock were sourced from various retail establishments across Sylhet District as shown in Figure 7. Swab samples from the meat were aseptically collected. All retail meat swab samples were pre-enriched by incubating in buffered peptone water medium (Oxoid, UK) at 37 °C for 24 h. Then, the pre-enriched culture medium was inoculated into Modified Semisolid Rappaport Vassiliadis (MSRV) medium enriched with novobiocin selective supplement (SR0181E) and incubated for 24 ± 3 h at 42 °C. Then the culture was streaked onto *Salmonella Shigella* agar plates (Sigma-Aldrich, Darmstadt, Germany) following appropriate methods. The presumptive positive colonies were subcultured onto xylose-lysine deoxycholate (XLD) agar medium. The suspected colonies of *Salmonella* were detected by their black-centered red hue characteristics on XLD agar plates (Oxoid, UK) [54,74,75]. The suspected colonies were further inoculated onto nutrient agar, followed by MacConkey agar (Oxoid, UK) plates, in accordance with the procedures outlined in the ISO 6579 manual as previously reported [76]. Biochemical tests, including Sugar Fermentation, Citrate, Methyl-Red-Voges Proskauer, Motility, Indole, and Urease tests, were conducted. Polymerase chain reactions (PCR) were performed to identify *Salmonella enterica* serovars and to screen for ESBL resistance genes as previously reported [77].

### 4.4. Identification of S. enterica Serovars and Detection of ESBL-Resistance Genes

DNA extraction was carried out using a DNA extraction kit, following the manufacturer’s instructions (AddBio Incorporated Limited, Daejeon, Republic of Korea). Table 4 lists the primers used for detecting *Salmonella enterica* serovars and ESBL-resistance genes as previously reported [74,77,78,79].

### 4.5. Antimicrobial Susceptibility Testing (AST)

*S.* Enteritidis and *S.* Typhimurium isolates were tested for antimicrobial susceptibility in vitro using the Kirby-Bauer disk diffusion method on Mueller-Hinton Agar plates, and results were interpreted according to the guidelines of the Clinical and Laboratory Standards Institute [80,81]. The AST was performed as previously reported [33] using 16 different antimicrobial disks (Oxoid, UK) belonging to 10 different antimicrobial categories including Penicillins: ampicillin (AMP, 10 μg); Tetracyclines: tetracycline (TE, 30 μg); Non-extended spectrum cephalosporins: cefuroxime (CXM, 30 μg); Extended-spectrum cephalosporins: ceftriaxone (CTR, 30 μg), ceftazidime (CAZ, 30 μg), cefotaxime (CTX, 30 μg); Quinolones: ciprofloxacin (CIP, 5 μg), nalidixic acid (NA, 30 μg); Macrolides: azithromycin (AZM, 15 μg); Folate pathway antagonists: trimethoprim- sulfamethoxazole (COT, 1.25/23.75 μg); Aminoglycosides: gentamicin (GEN, 10 μg), amikacin (AK, 30 μg); Monobactams: aztreonam (AT, 30 μg); Carbapenems: meropenem (MEM, 10 μg), imipenem (IMP, 10 μg); Phenicols: chloramphenicol (CL, 30 μg). The selection of the antibiotic disks in the present study was based on several prescriptions by local veterinarians targeting *Salmonella* infections in local cattle, goat, and chicken farms.

### 4.6. Molecular Detection of ESBL Genes

Multiplex polymerase chain reaction was performed as previously reported [82] to detect antimicrobial resistance genes, namely *bla*_TEM_, *bla*_SHV_, *bla*_OXA_*, bla*_CTX-M1_, *bla*_CTX-M2_, *bla*_CTX-M9_, *MultiCase*_ACC_, and *MultiCase*_DHA_, in antibiotic-resistant *Salmonella enterica* serovars Enteritidis and Typhimurium isolates. The PCR utilized specific oligonucleotide primers outlined in Table 4. The PCR amplification conditions included an initial denaturation at 95 °C for 5 min, followed by 30 cycles of 94 °C for 30 s, 62 °C for 90 s, and 72 °C for 60 s and also final extension at 72 °C for 10 min. Positive bands were visualized using agarose gel electrophoresis. Negative control (Nuclease free water) was used in all PCR reactions.

### 4.7. Determination of Multiple Antibiotic Resistance Index (MARI) and MDR

The MAR index was calculated and evaluated using the method described by [76] with the formula: MAR = The count of antimicrobial agents to which the isolate exhibits resistance divided by the number of antimicrobial agents tested. Resistance to one or more antimicrobial agent in three or more antimicrobial categories was defined as MDR (multidrug-resistance) as previously reported [83,84]. The MAR index of 0.20 or higher was deemed indicative of a high-risk source for bacterial contamination or substantial “resistance”.

### 4.8. Statistical Analysis

A univariate analysis was conducted using a Chi-squared test to assess the associations among various explanatory variables. Fisher’s Exact Test was conducted when more than 20% of the cells had an expected count below 5. *p* value < 0.05 considered as level of significance. Data analysis and visualization were conducted using SPSS version 26 (Version 26.0., IBM Corp., Armonk, NY, USA), GraphPad Prism 8.4.2 (GraphPad Software, Boston, MA, USA), and R 4.3.2 version.

### 4.9. Geospatial Mapping and Plotting

The geographical mapping of the study location was conducted using ArcGIS software (ArcMap 10.8, ESRI, Redlands, CA, USA), with shapefile data sourced from (www.diva-gis.org, accessed on 12 March 2024). These maps effectively portrayed choropleth representations, illustrating both the study area and the prevalence of key explanatory variables, alongside corresponding sample sizes. Additionally, a bi-directional bar (Mirror bar) diagram was employed to visually depict the prevalence of *Salmonella enterica* serovar Enteritidis and Typhimurium among livestock and poultry retail meat samples. To emphasize the antibiogram profile of the isolates, Origin-Pro 2024 (www.originlab.com, accessed on 14 March 2024) was used to generate heat maps with a dendrogram, providing a comprehensive overview of the data. This included heatmaps with hierarchical cluster analysis (HCA), illustrating the sensitivity pattern of antibiotics, with clustering depicted as a dendrogram. Additionally, a scatter plot correlation matrix with histograms was generated to display MARI value of *Salmonella enterica* serovars, utilizing Origin-Pro 2024 (www.originlab.com, accessed on 15 March 2024). For the portrayal of MDR and the antibiogram profile of the isolates, GraphPad Prism 8.4.2 (GraphPad Software, Boston, MA, USA) was utilized, presenting the data through a Stack bar diagram. The heatmap was created using OriginPro 2024 with “Heatmap with Dendrogram” packages, and the correlation plot was created using the “metan” package on R and RStudio 4.3.2 version.

## 5. Conclusions

The prevalence of *Salmonella* spp., particularly *S.* Enteritidis and *S.* Typhimurium, underscores the potential health risks associated with contaminated livestock-origin meat consumption. Notably, all *Salmonella* isolates in the present study from cattle meat samples demonstrated multidrug resistance, while a significant proportion of isolates from goat and chicken meat samples demonstrated complete resistance to multiple drugs. The high prevalence of ESBL genes, including *bla*_TEM_, *bla*_SHV_, *bla*_CTX-M-1_, and *bla*_OXA_, highlights the urgent need for enhanced surveillance and antimicrobial stewardship measures to mitigate the dissemination of antibiotic-resistant *Salmonella* serovars in raw meat, thus safeguarding public health. Moreover, the differential prevalence of ESBL resistance determinants detected in *Salmonella* serovars isolated from meat samples in the current study emphasizes the importance of implementing specific measures tailored to livestock production systems. Future investigations including genomic surveillance will help delineate the underlying antimicrobial resistance mechanisms in *Salmonella* serovars and will help provide insights to develop effective strategies to combat the extensive and multi-drug resistance and ESBL production in livestock-origin meat for human consumption. Effective intersectoral and interdisciplinary collaboration utilizing One health approach among healthcare authorities, veterinary professionals, environmental agencies, food producers, and consumers is critically required to better address the pressing problems of public safety and to reduce the continuous threats posed by the emergence and spread of antibiotic-resistant bacteria in the food distribution chain.

## Figures and Tables

**Table 1:** The prevalence of *S.* Typhimurium isolated from retail cattle, goat, and chicken meat samples obtained from 13 upazilas of Sylhet in the present study.

Category	Cattle Meat		Goat Meat		Chicken Meat		*p*-Value (Fisher’s Exact Test)
	x/N	Prevalence (%) 95% CI	x/N	Prevalence (%) 95% CI	x/N	Prevalence (%) 95% CI	
**Location**							
Balaganj	1/14	7.14% (0.18–33.87)	1/14	7.14% (0.18–33.87)	6/24	25.00% (9.77–46.71)	0.20
Bianibazar	2/12	16.67% (2.09–48.41)	1/8	12.50% (0.32–52.65)	3/17	17.65% (3.80–43.43)	0.95
Bishwanath	2/18	11.11% (1.37–34.71)	1/20	5.00% (0.13–24.87)	2/24	8.33% (1.03–27.00)	0.79
Companiganj	1/14	7.14% (0.18–33.87)	0/15	0%	1/18	5.56% (0.14–27.29)	0.61
Dakkhin Surma	2/21	9.52% (1.18–30.38)	1/17	5.88% (0.15–28.69)	3/21	14.29% (3.05–36.34)	0.68
Fenchuganj	2/13	15.38% (1.92–45.43)	3/13	23.08% (5.04–53.81)	3/20	15.00% (3.21–37.89)	0.82
Golapganj	2/15	13.33% (1.66–40.46)	1/16	6.25% (0.16–30.23)	2/14	14.29% (1.78–42.81)	0.74
Gowainghat	5/14	35.71% (12.76–64.86)	1/14	7.14% (0.18–33.87)	2/15	13.33% (1.66–40.46)	0.12
Jaintapur	2/13	15.38% (1.92–45.45)	3/12	25.00% (5.49–57.19)	3/17	17.65% (3.80–43.43)	0.79
Kanaighat	2/12	16.67% (2.09–48.41)	2/11	18.18% (2.28–51.78)	2/16	12.50% (1.55–38.35)	0.91
Osmaninagar	1/17	5.88% (0.15–28.69)	1/13	7.69% (0.19–36.03)	4/30	13.33% (3.76–30.35)	0.68
Sylhet Sadar	5/50	10.00% (3.33–21.81)	2/47	4.26% (0.52–14.54)	4/54	9.26% (3.08–20.30)	0.55
Zakiganj	4/12	33.33% (9.92–65.11)	2/10	20.00% (2.52–55.61)	3/15	20.00% (4.33–48.09)	0.68
Total	31/225	13.78% (9.56–18.98)	19/210	9.05% (5.54–13.77)	38/285	13.33% (9.61–17.84)	0.246 ^a^
		*p*-value = 0.390		*p*-value = 0.958		*p*-value = 0.85	

Fisher’s Exact Test, ^a^ Superscript means Chi-square test.

**Table 2:** The prevalence of *Salmonella* Enteritidis in different sub-districts (*n* = 13) of Sylhet, isolated from retail cattle, goat, and chicken meat in the present study.

Category	Cattle Meat		Goat Meat		Chicken Meat		*p*-Value (Fisher’s Exact Test)
	x/N	Prevalence (%) 95% CI	x/N	Prevalence (%) 95% CI	x/N	Prevalence (%) 95% CI	
**Location**							
Balaganj	1/14	7.14% (0.18–33.87)	1/14	7.14% (0.18–33.87)	5/24	20.83% (7.13–42.15)	0.35
Bianibazar	1/12	8.33% (0.21–38.48)	0/8	0%	5/17	29.41% (10.31–55.96)	0.12
Bishwanath	1/18	5.56% (0.14–27.29)	0/20	0%	5/24	20.83% (7.13–42.15)	0.06
Companiganj	2/14	14.29% (1.78–42.81)	1/15	6.67% (0.17–31.95)	3/18	16.67% (3.58–41.42)	0.68
Dakkhin Surma	5/21	23.81% (8.22–47.17)	0/17	0%	3/21	14.29% (3.05–36.34)	0.10
Fenchuganj	1/13	7.69% (0.19–36.03)	1/13	7.69% (0.19–36.03)	6/20	30.00% (11.89–54.28)	0.14
Golapganj	1/15	6.67% (0.17–31.95)	2/16	12.50% (1.55–38.35)	5/14	35.71% (12.76–64.86)	0.09
Gowainghat	2/14	14.29% (1.78–42.81)	0/14	0%	2/15	13.33% (1.66–40.46)	0.34
Jaintapur	2/13	15.38% (1.92–45.45)	2/12	16.67% (2.09–48.41)	4/17	23.53% (6.81–49.90)	0.83
Kanaighat	2/12	16.67% (2.09–48.41)	1/11	9.09% (0.23–41.28)	0/16	0	0.26
Osmaninagar	1/17	5.88% (0.15–28.69)	0/13	0%	3/30	10.00% (2.11–26.53)	0.48
Sylhet Sadar	1/50	2.00% (0.05–10.65)	1/47	2.13% (0.05–11.29)	9/54	16.67% (7.92–29.29)	0.004
Zakiganj	1/12	8.33% (0.21–38.48)	2/10	20.00% (2.52–55.61)	1/15	6.67% (0.17–31.95)	0.54
Total	21/225	9.33% (5.87–13.91)	11/210	5.24% (2.64–9.18)	51/285	17.89% (13.63–22.85)	<0.001 ^a^
		*p*-value = 0.501		*p*-value = 0.958		*p*-value = 0.85	

Fisher’s Exact Test, ^a^ Superscript means Chi-square test.

**Table 3:** The frequency of ESBL and β-lactam resistance genes in *Salmonella enterica* Serovars isolated from retail cattle, goat, and chicken meat in the present study.

Serovar	ESBL Class	ESBL Genes	Cattle Meat	Goat Meat	Chicken Meat	*p*-Value (χ^2^-Test/Fisher’s Exact Test
			Percent (Frequency)	Percent (Frequency)	Percent (Frequency)	
*S.* Enteritidis	Class-A	*bla*_TEM_	76.19% (16/21)	36.36% (4/11)	68.63% (35/51)	0.06 ^a^
		*bla*_SHV_	14.28% (3/21)	9.09% (1/11)	3.92% (2/51)	0.24
		*bla*_CTX-M-1_	0	0	15.69% (8/51)	0.07
		*bla*_CTX-M-2_	0	0	0	N/A
		*bla*_CTX-M-9_	0	0	3.92% (2/51)	0.53
	Class-C	*MultiCase*_ACC_	0	0	0	N/A
		*MultiCase*_DHA_	0	0	0	N/A
	Class-D	*bla*_OXA_	19.04% (4/21)	0	3.92% (2/51)	0.07
*S.* Typhimurium	Class-A	*bla*_TEM_	70.96% (22/31)	63.16% (12/19)	60.53% (23/38)	0.66 ^a^
		*bla*_SHV_	19.35% (6/31)	21.05% (4/19)	2.63% (1/38)	0.05
		*bla*_CTX-M-1_	0	0	7.89% (3/38)	0.23
		*bla*_CTX-M-2_	0	0	0	N/A
		*bla*_CTX-M-9_	0	0	0	N/A
	Class-C	*MultiCase*_ACC_	0	0	0	N/A
		*MultiCase*_DHA_	0	0	0	N/A
	Class-D	*bla*_OXA_	25.80% (8/31)	0	10.53% (4/38)	0.023

N/A: Not applicable, ^a^ refers to χ^2^ test.

**Table 4:** The primers used in the identification of ESBL and β-lactams resistance in *Salmonella enterica* serovars isolated from retail cattle, goat, and chicken meat in the present study.

Primers/Organisms	Primer Sequences	Amplicon Size (bp)	Reference
*invA/Salmonella* spp.	F-GTGAAATTATCGCCACGTTCGGGCAA	284	[77]
	R-TCATCGCACCGTCAAAGGAACC		
*sefA/S.* Enteritidis	F-GATACTGCTGAACGTAGAAGG	488	[79]
	R-GCGTAAATCAGCATCTGCAGTAGC		
*fliC/S.* Typhimurium	F-CGGTGTTGCCCAGGTTGGTAAT	620	[77]
	R-ACTGGTAAAGATGGCT		
*bla*_TEM_	F-CATTTCCGTGTCGCCCTTATTC	800	[78]
	R-CGTTCATCCATAGTTGCCTGAC		
*bla*_SHV_	F-AGCCGCTTGAGCAAATTAAAC	713	
	R-ATCCCGCAGATAAATCACCAC		
*bla*_OXA_	F-GGCACCAGATTCAACTTTCAAG	564	
	R-GACCCCAAGTTTCCTGTAAGTG		
*bla*_CTX-M1_	F-TTAGGAAATGTGCCGCTGTA	688	
	R-CGATATCGTTGGTGGTACCAT		
*bla*_CTX-M2_	F-CGTTAACGGCACGATGAC	404	
	R-CGATATCGTTGGTGGTACCAT		
*bla*_CTX-M9_	F-TCAAGCCTGCCGATCTGGT	561	
	R-TGATTCTCGCCGCTGAAG		
*MultiCase*_ACC_	F-CACCTCCAGCGACTTGTTAC	346	
	R-GTTAGCCAGCATCACGATCC		
*MultiCase*_DHA_	F-TGATGGCACAGCAGGATATTC	997	
	R-GCTTTGACTCTTTCGGTATTCG		

## Data Availability

The data supporting the findings of this study are included in the manuscript. Additional data are available from the corresponding authors upon reasonable request.

## Supplementary Materials

The following supporting information can be downloaded at: https://www.mdpi.com/article/10.3390/antibiotics13070586/s1, Table S1: Antibiogram profile of *S. enterica* Serovar Enteritidis isolated from cattle, goat and chicken meat samples in the present study; Table S2: Antibiogram profile of *S. enterica* Serovar Typhimurium isolated from cattle, goat and chicken meat samples in the present study.
