# Supplementary material for: Molecular Characterization of Multidrug-Resistant and Extended-Spectrum β-Lactamases-Producing Salmonella enterica Serovars Enteritidis and Typhimurium Isolated from Raw Meat in Retail Markets
Source: Antibiotics (Basel). 2024 Jun 24;13(7):586. doi: 10.3390/antibiotics13070586 (PMC11274296; doi:10.3390/antibiotics13070586)
Supplement: Supplementary file 1 [file antibiotics-13-00586-s001.zip › antibiotics-2976865-supplementary.pdf]

Supplementary Table S1. Antibiogram profile of *S. enterica* Serovar Enteritidis isolated from cattle, goat and chicken meat samples in the present study

**Antibiotics**	**Resistant (%)**	**Intermediate (%)**	**Sensitive (%)**
**AMP**	92.5	5	2.5
**GEN**	22.5	10	67.5
**AK**	12.5	2.5	85
**CXM**	60	17.5	22.5
**CTR**	0	0	100
**CTX**	45	0	55
**CAZ**	82.5	15	2.5
**TE**	87.5	2.5	10
**CIP**	15	25	60
**CL**	60	10	30
**AZM**	37.5	2.5	60
**COT**	55	0	45
**AZ**	35	12.5	52.5
**NA**	90	0	10
**IMP**	5	0	95
**MEM**	2.5	0	97.5

Supplementary Table S2. Antibiogram profile of *S. enterica* Serovar Typhimurium isolated from cattle, goat and chicken meat samples in the present study.

Antibiotics	Resistant (%)	Intermediate (%)	Sensitive (%)
**AMP**	100	0	0
**GEN**	0	1.72	98.28
**AK**	1.72	17.24	81.04
**CXM**	87.94	1.72	10.34
**CTR**	13.79	60.35	25.86
**CTX**	43.1	34.48	22.42
**CAZ**	98.28	1.72	0
**TE**	70.69	20.69	8.62
**CIP**	20.69	8.62	70.69
**CL**	84.48	6.9	8.62
**AZM**	31.03	5.17	63.8
**COT**	58.62	24.14	17.24
**AZ**	43.1	10.34	46.56
**NA**	94.83	0	5.17
**IMP**	3.45	0	96.55
**MEM**	5.17	0	94.83
